# Reliability and validity of the Chinese version of the Benefit Finding Questionnaire for people with mental disorders

**DOI:** 10.1371/journal.pone.0291586

**Published:** 2024-09-06

**Authors:** Xinyu Cao, Xiaomeng Tian, Yan Wen, Peijuan Li, Ruyu Ge, Xiaolin Li, Mei Feng

**Affiliations:** 1 Department of Pulmonary and Critical Care Medicine, West China Hospital, Sichuan University/West China School of Nursing, Sichuan University, Chengdu, China; 2 Department of Cardiology, West China Hospital, Sichuan University/West China School of Nursing, Sichuan University, Chengdu, China; 3 Department of Anesthesiology, West China Hospital, Sichuan University, Chengdu, China; 4 West China School of Nursing, Sichuan University/West China Hospital, Sichuan University, Chengdu, China; 5 Nursing Key Laboratory of Sichuan Province, China; COMSATS University Islamabad - Wah Campus, PAKISTAN

## Abstract

**Background:**

Benefit finding (BF) is correlated with mental health and recovery, and its presence will contribute to the recovery of patients with mental disorders. Most of the current tools for assessing BF in patients with somatic disorders are not adequate for patients with mental disorders. The present study proposes to introduce the Benefit Finding Questionnaire for People with Mental Disorders and to validate its psychometric properties.

**Methods:**

The Beaton translation model was used to translate and cross-culturally adjust the Japanese version of the Benefit Finding Questionnaire for People with Mental Disorders. A survey of 514 people with mental disorders was conducted from January 2022 to October 2022 using a general information questionnaire and a translated Chinese version of the Benefit Finding Questionnaire for People with Mental Disorders (BFQ-C) using a convenience sampling method. The quality of the questionnaire was examined in terms of item analysis, reliability, and validity.

**Results:**

The results of the item analysis showed that all items met the requirements. The interrater agreement of the BFQ-C was good, with an interrater agreement = 0.714; the values of the item-level content validity index ranged from 0.75 to 1.00; and the average of all item-level content validity index on the scale = 0.958. Exploratory factor analysis extracted three main factors “change in relationship with others,” “change in spirituality,” and “change in values and thinking styles”—and the cumulative variance contribution rate was 57.70%. The results of the confirmatory factor analysis were χ^2^/df of 2.194, Root Mean Square Error of Approximation of 0.075, and comparative fit index of 0.919, indicating that the model fitted well. The questionnaire had a Cronbach’ alpha of 0.936, a split reliability of 0.956, and a retest reliability of 0.939.

**Conclusion:**

The BFQ-C demonstrated good reliability and validity, and can be used to assess the BF level of people with mental disorders (e.g., anxiety disorders, depressive disorders, schizophrenia, and bipolar disorders) in China.

## Introduction

Mental disorders are a group of disorders in which mental activity (e.g., cognition, emotion, and volitional behavior) is impaired due to biological, psychological, social, or other undesirable factors, severely affecting social functioning and causing subjective distress. According to surveys, the prevalence of mental disorders is high, as approximately 1.1 billion people worldwide are affected by mental disorders or substance abuse disorders [1]. The lifetime prevalence of mental disorders can reach 22.1% in volatile regions [2]. The results of a survey in China showed that the lifetime prevalence of six types of mental disorders—mood disorders, anxiety disorders, alcohol-use and drug-use disorders, schizophrenia and other psychotic disorders, eating disorders, and impulse-control disorders)—was 16.6%, and one seventh of the population suffered from at least one mental disorder during their lifetime [3]. In addition, mental disorders can impose a serious burden on society, with the total medical costs of mental disorders accounting for more than 15% of China’s total health care costs and 1.1% of China’s gross domestic product [4].

Benefit finding (BF) is a key concept in positive psychology, with positive creativity, personality traits, emotions, and human cognitive experiential processes as its main areas of exploration [5]. Developed in the United States at the end of the 20th century, an early prototype was put forward by Taylor et al. in their proposed cognitive adaptation theory [6], according to which Tennen defined benefit discovery as a cognitive adaptation process in which an individual discovers personal, psychological, and social benefits from a negative life event [7]. Benefit finding in the field of disease refers to a number of positive changes that occur in a patient during the course of the disease [8]. Andresen et al. noted that BF is associated with fewer negative emotions and higher well-being indices, and that its initiation occurs at the very beginning of an individual’s recovery [9]. A meta-analysis showed that higher BF levels were associated with less depression, more positive attitudes towards life, and higher levels of well-being [10]. Sato et al. found that BF was one of the predictors of psychological well-being in Japanese patients with rheumatoid arthritis, and that BF was associated with emotional support and self-care [11]. Manne showed that BF levels were negatively associated with depressed mood in a survey of gynecological oncology patients [12]. Liu et al. found that significant increases in benefits were associated with decreases in depressive symptoms, and that there was a relationship between the experience of perceived cancer benefit findings and improvements in psychological functioning in cancer patients receiving psychotherapy [13]. A Japanese series of studies noted that knowledge of the presence of BF aided in the recovery of patients with psychiatric disorders, with individual recovery levels significantly correlating with BF levels, and higher BF levels in patients in later stages of recovery [14, 15]; these results suggest that researchers could reduce negative emotions in patients with psychiatric disorders by increasing their BF level, which would further promote individual recovery.

Currently, there are many versions of BF scales in China and abroad; however, there are few scales with clear and good reliability for BF in patients with mental disorders, and most of them are used to assess BF level in patients with physical illnesses. These scales include the Benefit Finding Scale (BFS) developed by Antoni for breast cancer patients [16], and adapted and further modified for other populations [17], and the Universal Post Traumatic Growth Rating Scale (UPGRS) developed by Tedeschi and Calhoun [18]. In addition, there are some disease-specific BF scales, such as the perceived benefits scale for cancer patients developed by Chien et al. [19], the Benefit Finding Scale for Multiple Sclerosis Patients developed by Pakenham and Cox [20], the General Population Perception of Benefit Scale for Disease-Free Populations developed by Cassidy et al. [21], the Childhood Illness Benefit Discovery Scale for children and parents of children developed by Phipps et al. [22] and Currier et al. [23], the Cancer Patient Benefit Discovery Scale for cancer patients developed by Ando et al. [24], and the Adolescent Benefit Finding Questionnaire for Adolescents with Chronic Diseases developed by von Rezori et al. [25].

The earliest studies related to BF in China also focused on breast cancer, and now mainly include those with breast cancer [26], cervical cancer [27, 28], colorectal cancer [29–32], lung cancer [33, 34], ovarian cancer [35], other cancers, diabetes mellitus [36], coronary artery disease [37, 38], infertility [39, 40], and caregivers for various types of chronic diseases [41–47], with varying BF levels having been found in these populations. Fewer studies have been conducted on BF in patients with mental disorders. The concept of positive psychology was recently introduced into the field of psychiatry. For example, Li et al. and Fan et al. [48, 49] used positive psychology to intervene with patients with schizophrenia under the theoretical guidance of positive psychology; they found that they could alleviate clinical symptoms, and improve positive psychological and social functioning in patients.

Due to the specificity of mental disorders, patients’ moods are often accompanied by continuous improvement or deterioration over time. The above-mentioned scales are not yet able to assess BF level in patients with mental disorders, highlighting a need for scales suitable for this purpose. According to the literature, the Benefit Finding Questionnaire (BFQ) [50] developed by Chiba et al. is more suitable for the assessment of BF in patients with mental disorders. The BFQ is based on qualitative research and literature review, and was developed *via* a rigorous process, with good reliability and validity (Cronbach’s α coefficient of 0.81–0.93). In addition, the two main dimensions of the scale—strengthening relationships with others and changes in personal life values—are in line with the direction of post-disease benefits for people with mental disorders (e.g., changes in relationships with others and changes in personal life values). The BFQ is based on a 5-point Likert scale ranging from 1 to 5, with 21 items. However, as this questionnaire has only been applied in a few foreign studies and has not yet been introduced in China, We will adapt the questionnaire and apply it to the Chinese population. In this study, we intend to create a Chinese version of the BFQ (BFQ-C), and test its reliability and validity to provide a suitable tool for the assessment of BF in Chinese patients with mental disorders.

## Materials and methods

### Study design

A cross-sectional design was used to evaluate the psychometric properties of the BFQ-C.

### Translation and cross-cultural adaptation process

**Fig 1:**
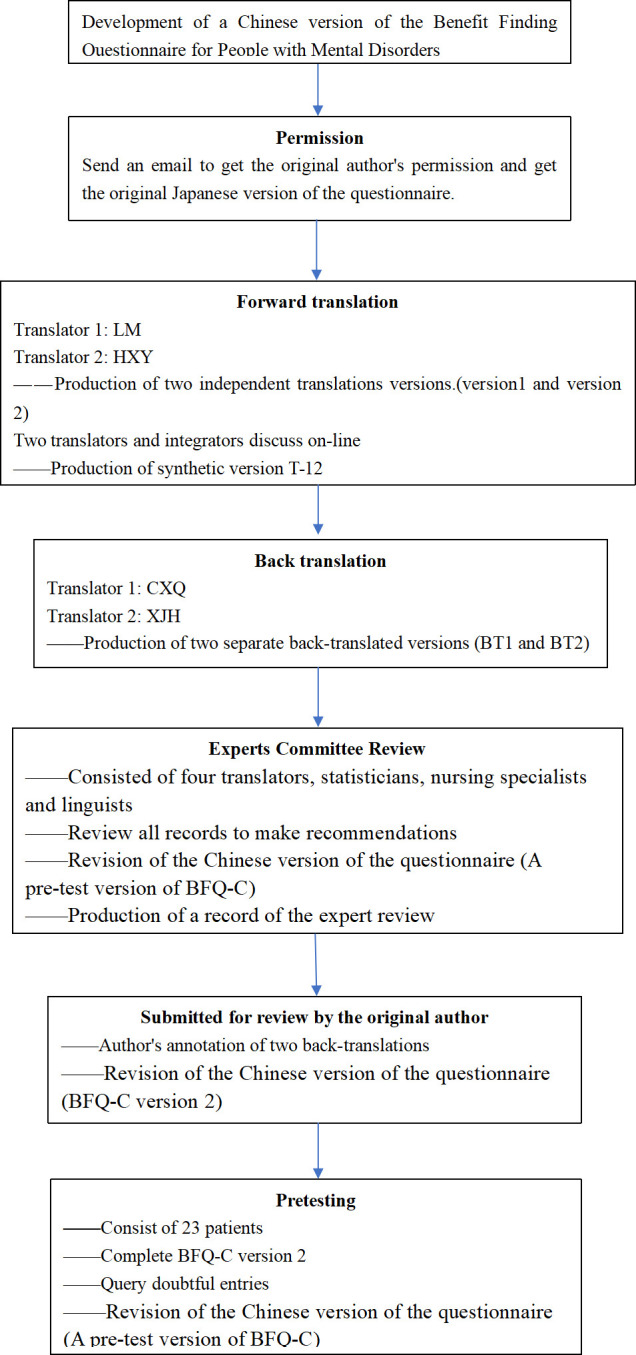
The process of developing the Chinese Benefit-Finding Questionnaire.

The Beaton translation model [51] was chosen for cross-cultural debugging of the BFQ, with the translation steps shown in Fig 1. We contacted the developer of the questionnaire, Rie Chiba, by email, explaining that the BFQ would be translated and adjusted to form a Chinese version of the questionnaire, and that scientific research would be conducted. The original Japanese version of the questionnaire was provided by him with his consent. Nursing professionals, experts with extensive experience in scale research, and nonmedical experts were invited to translate the original Japanese questionnaire under the guidance of the core concepts into a forward translation 1 (T1) and a forward translation 2 (T2). The experts were asked to write down areas of doubt during the translation process. The researcher and their group summarized the T1 and T2 versions, and comments to form the forward translation T-12 and carefully recorded the synthesis process and the handling of different translation comments in a written report. Two nonmedical professionals who were native Japanese speakers or who had studied in Japan and had not been exposed to the original scale were invited to independently retranslate the Chinese version of the T-12 into Japanese, resulting in back translation 1 (BT1) and back translation 2 (BT2). The expert committee comprised all translators, statisticians, caregivers, and linguists, seven in total The synthesis process was documented by the researcher based on the harmonization of all the information that existed in the process of translation and disagreement. A pretest version of the BFQ-C was created in the process. According to the Beaton translation model, the researcher submitted all written reports and versions to the original authors, who reviewed the results. The above process resulted in BFQ-C version 1.

### Pretesting

Twenty-three patients with mental disorders who fulfilled the inclusion and exclusion criteria were invited to participate in a face-to-face survey using the pretested version of the BFQ-C that was translated, retranslated, and reviewed by a panel of experts. The participants were mainly asked about any ambiguities and difficulties in understanding each item, and the BFQ-C version 2 was formed through the above process.

### Reliability and validity tests of the Benefit-Finding Questionnaire

In this study, item analysis, validity testing, and reliability testing were used to measure the quality of the BFQ-C. The item analysis included the critical ratio, correlation analysis, and Cronbach’s alpha coefficient. The validity testing included content validity and structural validity. Structural validity comprised two parts: exploratory factor analysis (EFA) and confirmatory factor analysis (CFA). The reliability testing comprised three methods: internal consistency, retest reliability, and split-half reliability. Retest reliability was chosen to be retested after two weeks.

### Participants and sample size

Convenience sampling was conducted from January 2022 to October 2022 in the inpatient psychiatric department of a tertiary general hospital. Data were collected using a one-to-one paper-based questionnaire. During the collection process, the investigator first screened out suitable subjects from the hospital’s electronic medical record system, and then asked them whether they were willing to participate in the study. The investigator fully explained the purpose and significance of the study to ensure voluntarily participation. Patients who did not participate in the study were assured that it would not have an impact on their treatment. According to the sample size calculation method for item analysis and exploratory factors, the sample size needed to be 5–10 times the number of items. Assuming a sampling error of 20%, the estimated sample size was 21 × (5 to 10) × (1 + 20%) = 126 to 252 cases. In the CFA stage, the minimum sample size should ideally be greater than 200 cases [52]; therefore, in this stage, a total minimum sample size of 452 cases was needed. Eventually, 514 patients were included in the study, of whom 300 were used for exploratory factor analysis in item screening, reliability testing, and validity testing, and 214 were used for CFA. The inclusion criteria for this study were as follows: patients with mental disorders diagnosed by psychiatrists in accordance with the 10th edition of the International Classification of Diseases, including patients with schizophrenia, depression, anxiety disorders, and bidirectional affective disorders, and other patients with psychiatric disorders; patients with psychiatric symptoms abated or mostly abated, and self-awareness recovered or mostly recovered according to the discharge plan to prepare for discharge before 1–2 days of remission; patients and their families provided informed consent and were willing to participate in the study. The exclusion criteria were as follows: patients with organic mental diseases and patients with combined serious physical diseases.

### Instruments

#### Participant characteristics

Demographic and disease-related information was obtained from the participants. Demographic data included age, sex, mode of payment for medical care, employment status, monthly household income, education level, place of residence, and marital status. Disease-related data included diagnosis, number of relapses, somatic comorbidities, mental comorbidities, and whether it is the first admission to a hospital for a psychiatric condition.

#### Chinese version of the Benefit Finding Questionnaire (BFQ-C)

The BFQ-C version 2 was formed as a result of the pre-survey and the determination of the original author’s back-translated version of this questionnaire. A total of 21 items were included in the questionnaire, which was scored on a 5-point Likert scale, with higher scores indicating a higher BF level.

### Statistical analysis

The statistical analysis was performed with IBM SPSS software (version 26.0). Descriptive statistics (frequencies, percentages, means and standard deviations) were used for sociodemographic and clinical variables.

For item analysis, first, the total scores of the BFQ-C were arranged in descending order; the top 27% was named the high group, and the bottom 27% was named the low group. Differences between the mean score of each item for the two groups were tested for statistical significance using the two independent samples *t*-test to delete the items with a decision value of <3 and *p*>0.05. The correlation coefficient between the score of each item and the total score of the questionnaire was analyzed; items with a correlation coefficient of <0.3 were considered for deletion. If the overall Cronbach’s α for the questionnaire increased after an item was removed, the item reduced the internal consistency of the questionnaire and was considered for deletion.

For validity testing, this study adopted the expert review method to evaluate the content validity of the BFQ-C. The item-level content validity index (I-CVI) is ≥0.78, and the average of all I-CVIs on the scale (S-CVI/Ave) is ≥0.9, indicating that the BFQ-C is acceptable. To test the structural validity of the BFQ-C, exploratory factor analysis (EFA) was carried out on the BFQ-C before conducting CFA, and the specific steps and judgement indexes were as follows: (i) EFA: In the EFA stage, the Kaiser-Meyer-Olkin (KMO) value and the Bartlett spherical test value were used to judge whether the data collection results of the BFQ-C were suitable for factor analysis. If the data result is a unit matrix, it is not suitable for factor analysis; if it is a unit matrix, it is not suitable for factor analysis, and when KM>0.9, it is considered very suitable for factor analysis. (ii) CFA [53]: the EFA model was subjected to CFA using Amos 25.0. There are many fit indexes for the model plots of CFA; chi-square/degree of freedom ratio (*χ2/df*), goodness-of-fit index (GFI), comparative fit index (CFI), and root mean square error of approximation (RMSEA) were used to evaluate the goodness of fit of the models in the present study [54].

Reliability was verified using internal consistency, retest reliability, and fold-half reliability, where a Cronbach α of ≥0.70 indicated good reliability and was an acceptable criterion. Pearson’s correlation coefficient was used to test retest reliability, which was done by selecting 30 patients who had previously participated in the study (with no significant changes in their conditions during the two-week period) to be reevaluated after two weeks [55]. The Spearman–Brown formula was used to calculate the split-half reliability of the BFQ-C by dividing all items into two groups using the parity method and calculating the correlation coefficient between the two, with higher correlation coefficients indicating better split-half reliabilities of the BFQ-C.

### Ethical considerations

This study followed the principles of voluntariness, confidentiality, fairness, and favorability, and was ethically approved by West China Hospital of Sichuan University under ethical approval number 2021 (929). The questionnaire was divided into three parts: (1) the purpose of the study and informed consent, where the research subjects needed to sign the informed consent form to participate in the study voluntarily; (2) the questionnaire, which the research subjects needed to complete according to their own actual situation; and (3) a final “thank you” from the researcher. Signed informed consent was required for this study; in the case of minors, the consent of their families was sought.

## Results

### Development of the Chinese version of the Benefit Finding Questionnaire

During the translation and cross-cultural debugging stage, amendments were made to items 3, 9, and 18, as follows: item 3 was amended from “I have gained the kind of trustworthy friends and companions that I would not have encountered if I had not suffered from a (mental) illness” to “I have gained trustworthy friends because of my illness”; in item 9, the word “trouble” was changed to “distress,” and in item 18, the words “your mental strength” were changed to “your mental strength compared to before the illness.”

Twenty-three patients (6 male and 17 female) were selected for the presurvey phase of the study, aged 14–39 years with a mean age of 21.22±7.42 years (see Table 1 for more detailed information). The cut-off criterion for the presurvey was when no patient had questions about the items. These 23 patients indicated that most of the topics were understandable but noted some awkward sentences or words, which were discussed and modified by the group and the linguist. All the documents from the translation process were sent again by the researcher to the original authors.

**Table 1:** Presurvey patients’ general information (n = 23).

Characteristics	Number of people	Constituent ratio (%)
**Gender**		
Male	6	26.1
Female	17	73.9
**Marital status**		
Unmarried	20	87
Married	3	13
**Education level**		
Junior high school and below	1	4.3
High school or technical secondary school	13	56.5
Bachelor’s degree or college	9	39.1
**Working state**		
Student	13	56.5
Stable / fixed work	4	17.4
Temporary work	1	4.3
No work	5	21.7

### Participant characteristics

A total of 300 subjects (104 male and 196 female) were studied in item analysis and reliability analysis stage phase, aged 13–77 years, with a mean age of 27.73 ± 13.97 years (see Table 2 for more detailed information).

**Table 2:** Sociodemographic and clinical characteristics of the participants (N = 300).

Characteristics	n (mean)	% (SD)
Age (years)	27.73±13.97	
**Gender**		
Male	104	34.7
Female	196	65.3
**Ethnicity**		
Han	280	93.3
others	20	6.7
**Education level**		
Junior high school and below	68	22.7
High school or technical secondary school	123	41
Bachelor’s degree or college	103	34.3
Bachelor’s degree or college	6	2
**Marital status**		
Unmarried	189	63
Married	100	33.3
Divorced or widowed	11	3.7
**Working state**		
Student	135	45
Stable / fixed work	81	27
Temporary work	16	5.3
No work	46	15.3
Retired	22	7.3
**Per capita monthly household income (RMB)**		
<2000	29	9.7
2001–4000	83	27.7
4001–6000	93	31
6001–8000	60	20
>8000	35	11.7
**Medical insurance**		
No	76	25.3
Yes	224	74.7
**Place of residence**		
Urban or town	233	77.7
Rural	67	22.3
**Diagnosis**		
Anxiety	66	22
Depression	106	35.3
Schizophrenia	51	17
Bipolar disorder	72	24
Others	5	1.7
Initial admission		
Yes	141	47
No	159	53
**Number of relapses**		
0–1	132	44
2–3	107	35.7
≥4	61	20.3
**Mental comorbidities**		
Yes	28	9.3
No	272	90.7
**Somatic comorbidities**		
Yes	51	17
No	249	83

#### Item analysis

Item-total correlation results are greater than 0.4 for all entries. And the critical ratio (CR) of all items was greater than 3 (7.048–22.829, *p*<0.05), meeting the requirements. Cronbach’s α of this questionnaire was 0.936; after removing each item, Cronbach’s α of the whole questionnaire did not become higher, and all items met the requirements, as shown in Table 3.

**Table 3:** Item analysis results of the Chinese version of Benefit Finding Questionnaire (BFQ-C) (n = 300).

Item	Item-total correlation(r)	CR	Cronbach’s Alpha if item deleted
1	0.528**	8.785**	0.935
2	0.482**	7.884**	0.936
3	0.479**	8.123**	0.936
4	0.563**	11.496**	0.935
5	0.627**	13.176**	0.934
6	0.493**	7.048**	0.936
7	0.656**	12.76**	0.933
8	0.589**	10.229**	0.934
9	0.568**	10.899**	0.935
10	0.794**	18.354**	0.930
11	0.718**	13.563**	0.932
12	0.662**	12.978**	0.933
13	0.679**	13.68**	0.933
14	0.785**	17.575**	0.930
15	0.792**	19.035**	0.930
16	0.717**	15.43**	0.932
17	0.708**	13.169**	0.932
18	0.726**	15.869**	0.932
19	0.823**	22.829**	0.930
20	0.780**	15.321**	0.931
21	0.674**	10.784**	0.933

CR, Critical Ratio. ***p < 0.001.

### Validity analyses

#### Content validity analysis

The content validity of the questionnaire was evaluated in this study using an expert consultation method with a panel of eight experts, including psychiatrists, mental health doctors and psychiatric nurses; specific information about the experts is shown in Table 4. The results showed that the interrater agreement of the BFQ-C was good, with IR = 0.714; I-CVIs ranged from 0.75 to 1.00, and S-CVI/Ave = 0.958. In this study, the I-CVI for the fifth item, “The opportunities to greet and speak to neighbors and people in the community have been,” was lower than 0.78, with low validity. Therefore, we referred to the EFA results as to whether this item should be retained, as shown in Table 5.

**Table 4:** Basic information on experts.

Expert committee	Age	Gender	Education level	Major	Years of professional experience
1	41	Female	Doctor degree	Cognitive and Clinical Psychology	17
2	35	Female	Doctor degree	Psychological Counseling and Nursing Management	10
3	53	Female	Bachelor’s degree	Psychiatric Nursing	35
4	39	Female	Doctor degree	Psychiatric Nursing	13
5	42	Male	Doctor degree	Psychiatric Nursing	9
6	33	Male	Master degree	Psychiatric Nursing	11
7	49	Female	Bachelor’s degree	Psychiatric Nursing	28
8	48	Male	Doctor degree	Psychosomatic Medicine and Scale Research	20

**Table 5:** Content validity index of the scale (n = 8).

Item	Expert1	Expert2	Expert3	Expert4	Expert5	Expert6	Expert7	Expert8	Number of experts with a rating of 3 or 4	I-CVI
1	3	3	4	4	4	4	4	2	7	0.875
2	3	3	3	4	4	4	4	2	7	0.875
3	3	4	3	4	4	4	4	4	8	1
4	3	4	3	4	4	4	4	2	7	0.875
5	2	3	2	3	4	4	3	3	6	0.75
6	3	4	3	4	4	4	4	4	8	1
7	3	4	3	4	4	4	4	3	8	1
8	3	4	4	4	4	4	4	2	7	0.875
9	2	3	4	4	4	4	4	3	7	0.875
10	4	4	4	4	4	4	4	4	8	1
11	3	4	3	4	4	4	4	4	8	1
12	3	4	3	4	4	4	4	4	8	1
13	4	4	3	4	4	4	4	4	8	1
14	4	4	3	4	4	4	4	4	8	1
15	4	4	3	4	4	4	4	4	8	1
16	4	4	3	4	4	4	4	4	8	1
17	4	4	3	3	4	4	4	4	8	1
18	3	4	3	4	4	4	4	4	8	1
19	4	4	3	4	4	4	4	4	8	1
20	4	4	3	4	4	4	4	4	8	1
21	4	4	4	4	4	4	4	4	8	1

#### Structural validity

*Exploratory factor analysis*. The results showed that the Kaiser-Meyer-O1kin value was 0.939 (i.e., >0.9), and the approximate chi-squared value of Bartlett’s sphericity test was 3390.61 (*p* < 0.001). Both of the above results indicated that it was appropriate to perform the factor analysis. Principal component analysis and varimax rotation extracted three factors, which explained 53.68% of the total variance. The rotated factor loading matrix for each item is shown in Table 6. The BFQ-C comprised three factors: “Change in values and thinking,” comprising items 10, 11, 12, 13, 14, 15, 16, 17, 18, 19, 20, and 21; “Changes in spirituality,” comprising items 3, 4, 7, 8, and 9; and “Changes in relationships with others,” comprising items 1, 2, and 6. The loadings of item 5 on factors 1 and 3 were 0.426 and 0.412, respectively. According to the results of the expert consultation on content validity, it was decided to delete item 5, as shown in Table 6.

**Table 6:** Factor analysis and mean scores of the BFQ-C.

Item	Mean	SD	Factor loading		
			Factor 1	Factor 2	Factor 3
1	3.16	1.054	0.236	0.131	**0.698**
2	2.76	1.075	0.031	0.349	**0.727**
3	2.41	1.013	0.037	**0.718**	0.271
4	2.79	1.028	0.257	**0.597**	0.191
5	2.49	1.003	0.426	0.285	0.412
6	3.2	1.017	0.275	0.07	**0.596**
7	2.54	1.045	0.317	**0.673**	0.226
8	2.87	1.047	0.435	**0.456**	0.066
9	2.61	1.147	0.264	**0.724**	0.029
10	2.95	1.198	**0.724**	0.222	0.31
11	2.77	1.115	**0.722**	0.285	0.033
12	2.72	1.073	**0.6**	0.327	0.083
13	2.77	1.157	**0.643**	0.332	0.036
14	2.88	1.244	**0.79**	0.197	0.188
15	2.88	1.116	**0.705**	0.254	0.314
16	2.47	1.068	**0.654**	0.398	0.043
17	2.97	1.127	**0.706**	0.095	0.275
18	2.68	1.29	**0.693**	0.135	0.294
19	2.61	1.144	**0.741**	0.359	0.188
20	2.91	1.078	**0.771**	0.167	0.247
21	3.05	1.126	**0.665**	-0.022	0.405

**Fig 2:**
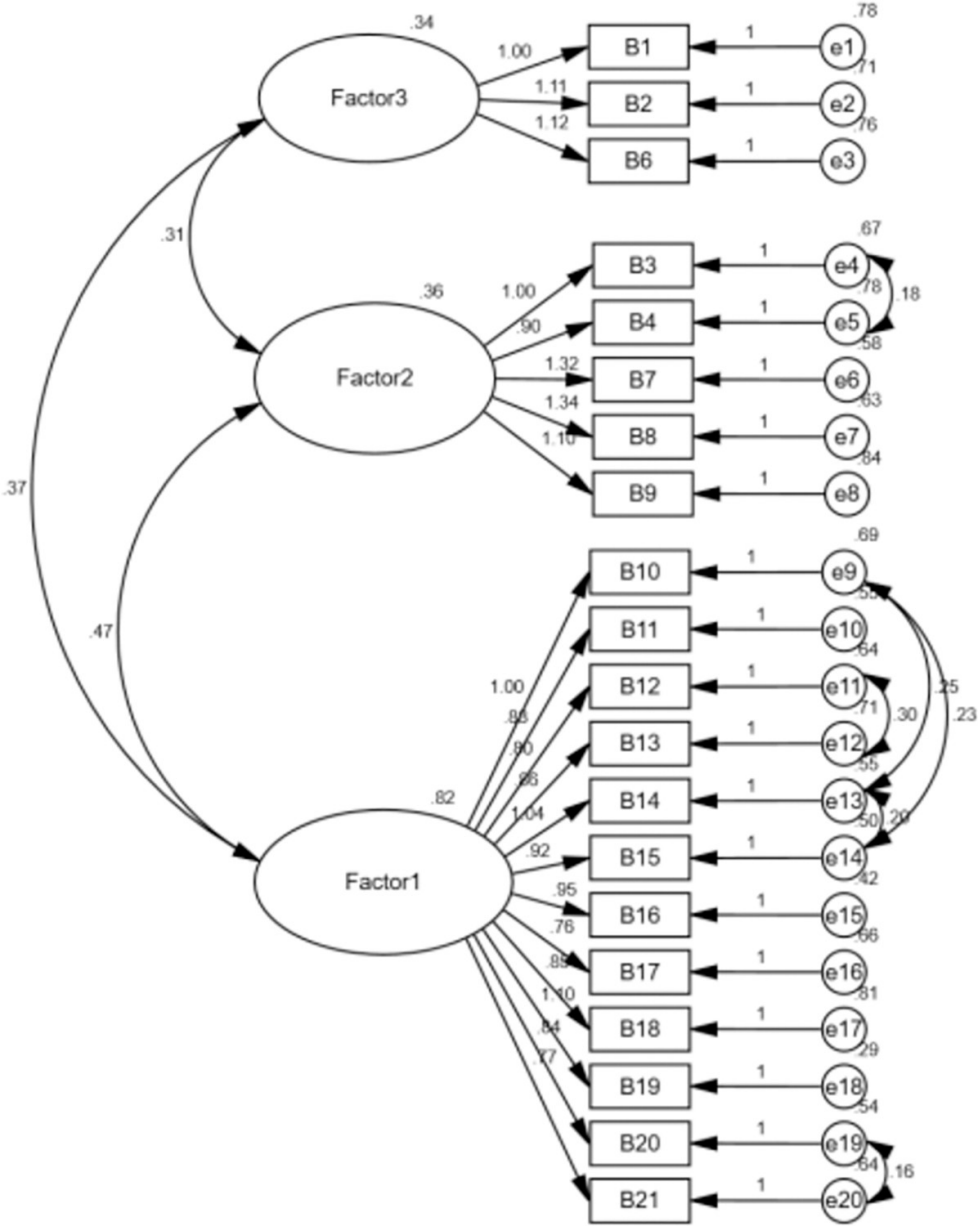
Results of the validation factor analysis of the Chinese Benefit Finding Questionnaire.

*Confirmatory factor analysis*. A further 214 questionnaires were used to validate the fit of the three-factor model derived from the EFA. The model contained three factors, between which a correlation was found, indicated by double arrows in the model assumptions. The model contained a total of 20 observed variables, each of which was affiliated with only one factor. Measurement residuals were assumed to exist for each observed variable, and the residuals were set to be independent of each other. To determine the units of the three latent variables, the study adopted the method of fixed factor loading by setting the dependent loadings of the first observed variable under each latent variable to 1. According to the suggestion of the correction index, the paths between the residuals e4 and e5, e19 and e20, e13 and e14, e11 and e12, e9 and e13, and e9 and e14 were increased to improve the fitting results, as shown in Fig 2. The results showed that the model was still acceptable, with a chi-square/degree of freedom ratio (x^2^/df) of 2.194 (i.e., ≤3), an RMSEA of 0.075 (i.e., <0.08), and a CFI of 0.919 (i.e., >0.9) as shown in Table 7.

**Table 7:** Range of values and results of structural equation model fitting indicators.

Fitness index	*χ* ^*2*^ */df*	IFI	TLI	CFI	RMSEA
value range	≤3	>0.9	>0.9	>0.9	<0.08
Results	2.194	0.920	0.904	0.919	0.075

*Reliability analysis*. Cronbach’s alpha for the questionnaire was 0.936. Cronbach’s alpha for dimension 1, “Change in relationship with others,” dimension 2, “Change in spirituality,” and dimension 3, “Change in values and way of thinking was 0.631, 0.771, and 0.936, respectively. The items of the questionnaire were divided into two halves according to parity; correlation analysis of these two halves yielded a folded-half reliability of 0.956 for the questionnaire. The retest reliability after two weeks for the 30 patients was 0.939.

## Discussion

### Cross-cultural adaptation of the Chinese version of the Benefits Finding Questionnaire

The process of Chinese translation of this questionnaire strictly followed the steps of Beaton’s translation model [51]. During the translation and cross-cultural adaptation stage of the questionnaire, which coincided with the COVID-19 epidemic, due to closure and control measures, a large-scale gathering and discussion could not be conducted. This study adopted a flexible approach to the translation; after the completion of the forward translation, the researcher herself and her group integrated the two results, and then carried out a back translation. After this step, the original authors were invited to control the results of our translations. The original authors were also consulted after the first translation; they provided great help and support, and acknowledged the results of our study.

### Psychometric properties of the Chinese version of the Benefits Finding Questionnaire

The results of the correlation analysis showed that the correlation coefficient between the score of each item and the total score of the questionnaire were all greater than 0.4, and *p*<0.05, which met the requirements. The results of the critical ratio test showed that all entries met the requirements, with critical ratios all greater than 3.00, and *p*<0.05. As Cronbach’s alpha for the questionnaire did not become higher after deleting any of the items, all items met the requirement in this stage of item analysis.

In terms of content validity, the questionnaire showed good interrater agreement with IR = 0.714, I-CVIs ranging from 0.75 to 1.00, and S-CVI/Ave = 0.958; however, the I-CVI of the fifth item of the questionnaire, “The opportunities to greet and speak to neighbors and people in the community have been", was lower than 0.78, indicating low validity.

The questionnaire proved to have good structural validity. The KMO value of the questionnaire was 0.939, and the approximate chi-square value of Bartlett’s sphericity test was 3390.61 (*p* < 0.001), all of which meeting the conditions for conducting EFA. The EFA extracted a total of three factors with a cumulative ANOVA contribution of 57.70%, and the factor loadings for all items were greater than 0.4. The loadings of item 5 on factor 1 and factor 3 were 0.426 and 0.412, respectively. Combined with the results of the expert consultation on the content validity of item 5, the decision was made to delete item 5. Due to the development of society and the increase in work pressure, the general population would have little chance to greet their neighbors or people in the same neighborhood; therefore, item 5 is not quite in line with the habits of the majority of people in China. The EFA results differed slightly from the factors of the original questionnaire, the BFQ-C includes three factors, i.e., the first factor of the BFQ is divided into two factors. The reason for this was that the three factors were statistically derived and may have influenced by cultural differences leading to different understanding of the items by the patients. To further verify the structural validity of the BFQ-C, the remaining 214 data points were taken for CFA. The results of the CFA showed that by increasing the correlation between some of the residuals, all the fitting indexes were able to meet the requirements, which indicated that the questionnaire had good structural validity.

The reliability evaluation results showed that the BFQ-C had a Cronbach’s alpha of 0.936, a folded-half reliability of 0.956, and a retest reliability of 0.939, which indicated that the questionnaire had good reliability, stability, and consistency. However, Cronbach’s alpha for factor 1 of the questionnaire was 0.631, representing a medium level. There are two reasons for this result. First, the number of items in this dimension is small at only three. Second, due to cultural differences, items 1, 2, and 6 in dimension 1 may have lower Cronbach alphas due to different interpretations by the respondents. However, this entry was discussed by the Expert Committee and is more responsive to the benefit finding for persons with mental disorders and is therefore retained.

### Limitations

This study did have some limitations. Due to the impact of the COVID-19 epidemic, the study included psychiatric patients from a large tertiary care hospital, possibly introducing sample population bias. For example, women and young people are overrepresented in the sample. Therefore, it is recommended to validate the study in a wider range of patients in the future. As there is no “gold standard” in the field of psychiatric disorders for measuring BF in people with psychiatric disorders, calibration validity was not performed in this study. In addition, the sample for this study was selected from inpatient psychiatric patients and did not involve patients with mental disorders from the general community. Therefore, it is recommended that future studies include patients with mental disorders from the general community to validate this population.

## Conclusion

The introduction process of the BFQ strictly followed the Beaton translation model. The Chinese version of the Benefits Finding Questionnaire (BFQ-C) was finally formed after translation at the text level, expert consultation, and data analysis, with good reliability and validity. The questionnaire comprised three factors—change in values and thinking, change in spirituality, and change in relationship with others—with a total score of 20–100, with higher scores representing higher BF levels. The questionnaire had a Cronbach alpha of 0.936, a folded-half reliability of 0.956, and a retest reliability of 0.939. In summary, the BFQ-C can be used to assess BF level in those with mental disorders (e.g., anxiety disorders, depressive disorders, schizophrenia, and bipolar disorders) in China.

## Supporting information

S1 File A file includes the Japanese, English and Chinese versions of the Benefit Finding Questionnaire. (RAR)

S2 File A file that contains all the data needed to arrive at the results of this study, on which the results of this study can be obtained. (XLSX)
